# 3′,4′-Dimethoxyflavone and valproic acid promotes the proliferation of human hematopoietic stem cells

**DOI:** 10.1186/scrt208

**Published:** 2013-05-24

**Authors:** Kiranpreet Kaur, Mohammad Reza Mirlashari, Gunnar Kvalheim, Jens Kjeldsen-Kragh

**Affiliations:** 1 Department of Immunology and Transfusion Medicine, Oslo University Hospital, Ullevål, Oslo, Norway; 2 Department of Cellular Therapy, Oslo University Hospital, Radiumhospitalet, Oslo, Norway

## Abstract

**Introduction:**

Human hematopoietic stem cells (HSCs) have been clinically used for transplantation and gene and cellular therapy for more than 4 decades. However, this use is limited because of the challenges in the *ex vivo* culturing of HSCs. The major hurdle is to amplify these cells without losing their self-renewing property.

**Methods:**

In our study, we tested 3′,4′-dimethoxyflavone (3′4′-DMF) and valproic acid (VPA) on the *ex vivo* expansion of HSCs under both normoxic (20% O_2_) and hypoxic (1% O_2_) conditions. 3′4′-DMF is a widely used anticancer drug that acts as a competitive antagonist of the aryl hydrocarbon receptor. VPA is a potent inhibitor of histone deacetylase and is used in the treatment of neurologic disorders.

**Results:**

Culturing HSCs (from mobilized peripheral blood) under normoxia, with 3′4′-DMF and VPA, highly preserved the CD34 positivity (3′4′-DMF, 22.1%, VPA, 20.3%) after 1 week and strongly enhanced the CD34^+^ cells (3′4′-DMF, 27.8 fold; VPA, 34.1 fold) compared with the control cultures (11.6% and 14.4 fold). Addition of 3′4′-DMF and VPA also resulted in more primary colonies and replating efficiency compared with control cultures. Although no significant effect was observed on the enhancement of CD34^+^ cells under hypoxia, the number of primary colonies was significantly higher than the control cultures.

**Conclusions:**

Based on these findings, this study presents, for the first time, *in vitro* evidence for a new and relevant effect of 3′4′-DMF on human HSCs. In addition, the results suggest a potential clinical use of 3′4′-DMF and VPA in HSC therapy.

## Introduction

*Ex vivo* expansion of human hematopoietic stem cells (HSCs) is a major challenge in cell therapy. Although advances have been made in understanding the role of various growth factors and cytokines that leads to the progressive maturation of various cell lineages, little is known about the factors that govern the self-renewal and primitive nature of HSCs. Recent attempts are focused on the identification of growth factors and pharmacologic agents to manipulate HSCs *in vitro*, with the aim to expand HSCs *ex vivo*, while maintaining their primary functional characteristics.

In our study, we tested a 3′,4′-dimethoxyflavone (3′4′-DMF) on the *ex vivo* expansion of HSCs. 3′4′-DMF is a competitive antagonist of the aryl hydrocarbon receptor (AhR) that inhibits AhR-mediated induction of cytochrome P450 1A1 [1]. The compound blocks transformation of the cytosolic AhR complex and formation of nuclear AhR complexes. 3′4′-DMF has extensively been used as an anticancer drug in various cancers (for example, breast cancer, leukemia, and oral cancer) [1-3]. However, nothing is known about the potential role of this compound in the expansion and differentiation of HSCs. The selection of 3′4′-DMF was based on the earlier studies showing the expression of AhR in HSCs [4]. The AHR is mainly a ligand-activated transcription factor responsible for the induction of drug-metabolizing enzymes. In addition, it has been suggested that AHR plays an important role in regulating hematopoiesis, for example, in HES-1-, c-MYC-, β-catenin-, and STAT5- dependent processes [4]. Moreover, treatment of donor mice with the AhR agonist dioxin, 2,3,7,8-tetracholorodibenzo-*p*-dioxin (TCDD), leads to decrease in the reconstitution activity of Lin^-^cKit^+^Sca^-^1^+^ cells in the recipient mice [5,6]. All these findings allude to the potential role of AHR in HSCs. Recently Boitano and co-workers [7] demonstrated the positive effect of a purine derivative, Stem Regenin (SR1), in the *ex vivo* expansion of mobilized peripheral blood-derived CD34^+^ cells by 50 fold [7]. SR1 was shown to act by antagonizing the AhR. Based on these fascinating findings, 3′4′-DMF was selected, that also acts by inhibiting the AhR. The effect of 3′4′-DMF on the proliferation, survival, and differentiation of CD34^+^ cells was determined both under normoxic (20% O_2_) and hypoxic (1% O_2_) conditions.

Another pharmacologic agent used in the present study was valproic acid (VPA). Histone deacetylase (HDAC) inhibitors (for example, VPA) have been successfully used for more than 2 decades, for the treatment of neurodegenerative disorders. VPA has been used as a first-line treatment drug for bipolar disorders. VPA helps in protecting against apoptosis insults both *in vitro* and *in vivo*[8]. Pretreatment with VPA protects cultured brain neurons from glutamate-induced apoptosis [9]. It has also been shown to display beneficial effects in cellular and animal models of neurodegenerative diseases such as stroke, Alzheimer disease, Parkinson disease, and spinal cord injury [10].

Although the role of HDAC inhibitors in regulating the growth, differentiation, and apoptosis in various tumors has been documented very well, just a handful of reports are available suggesting their potential use in stem cell biology. Moreover, these studies reported the effect of VPA on HSCs only under normoxic (20% O_2_) conditions. Culturing under hypoxic conditions was chosen because HSCs reside in specialized hypoxic niches in the bone marrow (BM). Besides, an extensive number of studies in the literature have shown that hypoxic *ex vivo* culture of BM or primitive hematopoietic progenitors results in the maintenance of the primitive phenotype and cell-cycle quiescence [11,12]. In addition, culturing of HSCs under low oxygen tension enhances the proliferation of HSCs and maintenance of SCID-repopulating cells more than under normoxic conditions [13].

## Materials and methods

### Cell source

The study protocol adhered to the guidelines of the Declaration of Helsinki and was approved by The Regional Committees for Medical and Health Research Ethics (Reference number 2010/510). Mobilized peripheral blood was collected from healthy donors, after informed consent, at the Department of Cellular Therapy, Oslo University Hospital. CD34^+^ cells were isolated from leukophoresis blood by using CD34 magnetic micro beads (Miltenyi Biotec, Germany) and MACS separation column. Isolated cells were tested for purity by using flow cytometry. For all isolations, the purity of CD34^+^ cells was 90% to 95%. The CD34^+^ cells were frozen in medium with 10% dimethyl sulfoxide (DMSO) and maintained in the vapor phase of liquid nitrogen (−180°C) until use.

### Phenotypic characterization of isolated CD34^+^ cells with flow cytometry

The total number and the percentage of viable cells were counted by Nucleocounter Chemometec (Allerød, Denmark) according to manufacturer’s manual. Three-color flow cytometry was performed to study the expression of cell-surface antigens (CD34, CD38, CD90, CD19, CD7, CD15, CD71, CD33, CD61) of CD34^+^ cells on days 0 and 7. All monoclonal antibodies and the cell-viability marker, 7-AAD, were obtained from BD Pharmingen (San Jose, CA, USA). Anti-CD38-PC5 was purchased from Beckman Coulter (Beckman Coulter, Miami, FL, USA).

### *Ex vivo* expansion of CD34^+^ cells

CD34^+^ cells (15 × 10^3^ cells/ml) were cultured in 12-well flat-bottomed culture plates. The cells were cultured in Cell Gro medium (Cell Genix, Freiburg, Germany) supplemented with a cocktail of five cytokines: Thrombopoietin (100 ng/ml; Cell Genix), Stem cell factor (100 ng/ml, Cell Genix), Flt3L (100 ng/ml; Cell Genix), interleukin 3 (20 ng/ml, Cell Genix), and interleukin 6 (20 ng/ml, Cell Genix). 3′,4′-Dimethoxy flavone (2.5 μ*M*, Sigma-Aldrich, St. Louis, MO, USA) and valproic acid (0.065 m*M*, Sigma-Aldrich) were added to the tested cultures along with cytokines. The normoxic cultures were incubated at 37°C and 5% CO_2_ for 7 days. The hypoxic cultures were maintained at 37°C and 1% O_2_ for 7 days. The cultures were supplemented with fresh cytokines at day 4. The total number of nucleated cells, viability, and phenotypic profiling were evaluated at day 7.

### Cell-cycle analysis

For cell-cycle analysis with flow cytometry, 1 × 10^5^ cells were harvested and washed with PBS, resuspended, and fixed by slow addition of 1 ml 100% ice-cold methanol to the mixer and stored at −20°C until analysis. After fixation, the cells were pelleted, washed with cold PBS, and then stained with 1.5 μg/ml Hoechst 33258 for 1 hour at 25°C in the dark. Ten thousand cells were analyzed with flow cytometry.

### Colonogenic assay

CD34^+^ cells were plated in Methocult (Stem Cell Technologies, Vancouver, BC, Canada), supplemented with IMDM (Stem Cell Technologies) and 10% FBS (Stem Cell Technologies). 3′4′-DMF and VPA were added to the Methocult before mixing it with the cell suspension in IMDM. One thousand cells suspended in 1 ml of prepared mixture were plated in a 35-mm dish in triplicate and cultured for 14 days at 37°C. The colonogenic assay was performed under normoxic (20% O_2_) conditions. Because culturing under hypoxia results in cell-cycle arrest, the colonogenic assay was performed under normoxic conditions only.

Colonies were counted under the phase-contrast microscope and scored into three categories: pure erythroid (burst-forming unit (BFU-E)), myelomonocytic (granulocyte-macrophage colony-forming unit (CFU-GM)), and mixed colony-forming unit (CFU-GEMM- granulocyte, erythroid, macrophage, megakaryocyte).

### Replating efficiency

The replating efficiency of the primary colonies was evaluated as described by De Felice *et al*. [14]. In brief, the primary colonies (erythroid, granulocyte-macrophage, and mixed colonies) were picked up individually from the 35-mm petri plate after 14 days of incubation. Each individual colony was dispersed into single-cell suspension in 200 μl of methylcellulose medium (Methocult) and seeded in separate wells of a 96-well plate. The replating efficiency was calculated as a percentage of colonies formed by seeded cells after 14 days of incubation under normoxia.

### Statistical methods

Data are presented as mean ± SD. The Student *t* test was used for significance testing. Differences were considered significant at *P* < 0.05.

## Results

### Effect of 3′4′-DMF and VPA on the *ex vivo* expansion of CD34^+^ cells under normoxia (20% O_2_)

**Figure 1:**
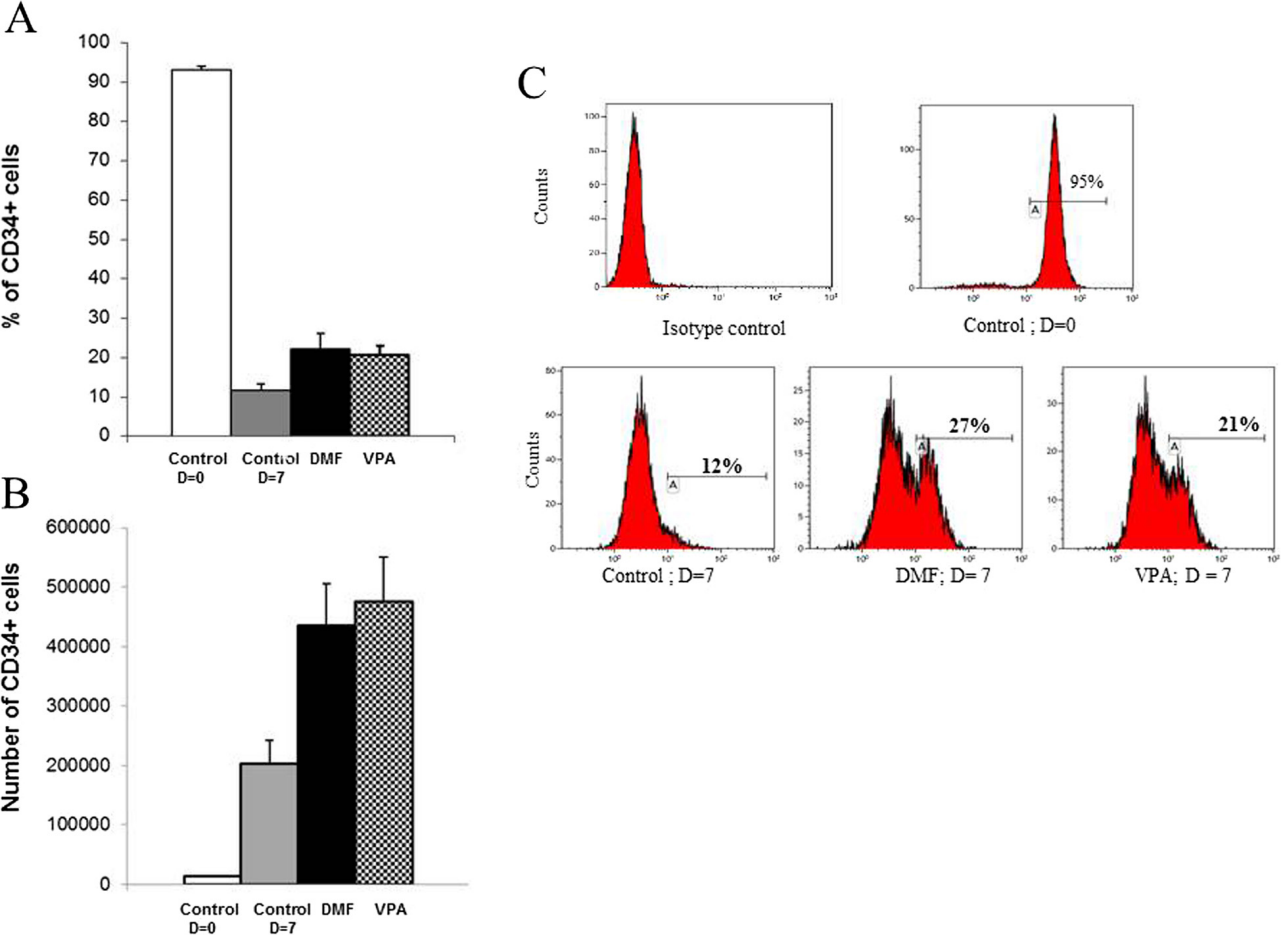
**3′4′-Dimethoxyflavone (DMF) and valproic acid (VPA) enhance the proportion of CD34**^**+ **^**cells.** The mean number and percentage of CD34^+^ cells was evaluated before (day 0) and after (day 7) the initiation of cytokine-induced cultures. (**A)** More mean CD34^+^ cells were found in the cultures with 3′4′-DMF and VPA compared with the control cultures after 7 days of amplification. (**B):** A higher percentage was found in the cultures with 3′4′-DMF and VPA compared with the control cultures after 7 days of amplification. **(C)** FACS analysis plots of CD34^+^ fractions from representative samples before (day 0) and after 7 days of amplification with cytokines alone (control), with 3′4′-DMF + cytokines and with VPA + cytokines. 3′4′-DMF (), VPA (), control at day 0() and control at day 7 (). The columns represent the mean ± SD values from three independent experiments.

The effect of 3′4′-DMF and VPA on the cytokine-induced *ex vivo* amplification of CD34^+^ cells was determined after 7 days of culture (Figure 1). Phenotypic analysis showed a significant increase in the total number as well as percentage of CD34^+^ cells in the cultures treated with 3′4′-DMF + cytokines (435,626.7 ± 69,577; 22.1% ± 2.7; *P* < 0.007) and VPA + cytokines (475,980 ± 73,927; 20.3% ± 2.2; *P* = 0.012) compared with the control cultures treated with cytokines alone (203,246.7 ± 39,222; 11.6% ± 1.4).

However, the percentages of CD34^+^CD90^+^ cells (3′4′-DMF + cytokines = 0.6% ± 0.16, VPA + cytokines = 0.7% ± 0.22, control at day 7 = 0.6% ± 0.08 and control at day 0 = 0.7% ± 0.2) and CD34^+^ CD38^-^ cells (3′4′-DMF + cytokines = 1.4% ± 0.16, VPA + cytokines = 1.5% ± 0.26, control at day 7 = 1.4% ± 0.16 and control at day 0 = 1.6% ± 0.2) did not differ between cultures treated with 3′4′-DMF or VPA in comparison with the control cultures.

**Figure 2:**
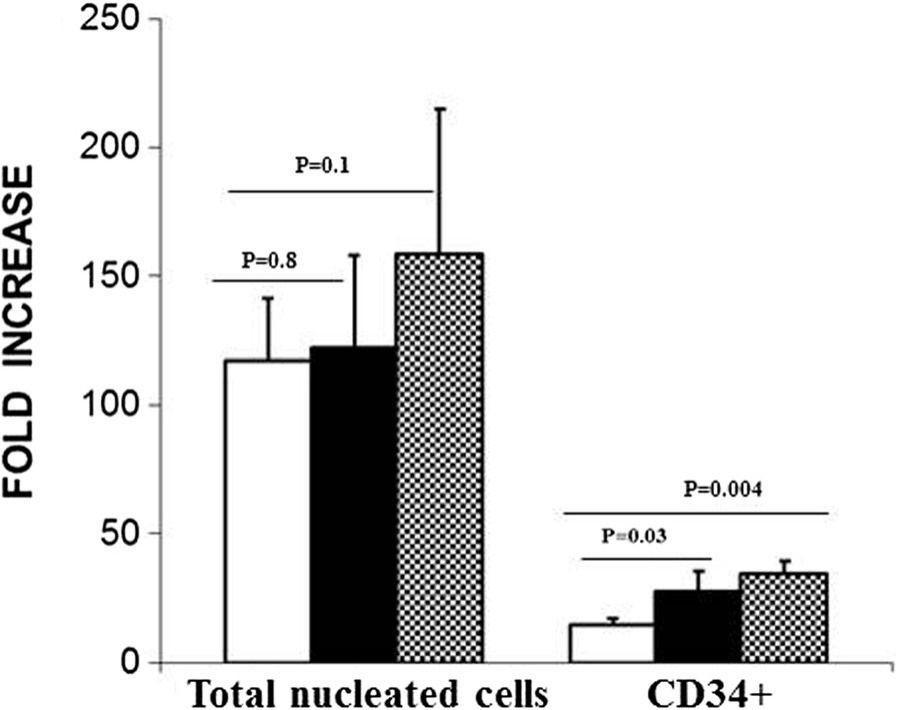
**3′4′-Dimethoxyflavone (DMF) and valproic acid (VPA) increased the proliferation of CD34**^**+ **^**cells in 7-day culture.** The effect of 3′4′-DMF and VPA on the amplification of TNC and CD34^+^ cells was evaluated as fold increase over the input (day 0) values. 3′4′-DMF (), VPA (), control (). The columns represent the mean ± SD values from three independent experiments.

Next we evaluated expansion of CD34^+^ cells by dividing the total number of cells obtained at day 7 to the total number of cells at day 0. As shown in Figure 2, no significant difference (*P* > 0.05) was found in the proliferation of total nucleated cells (TNCs) cells in the cultures treated with 3′4′-DMF + cytokines (121 fold) and VPA + cytokines (158 fold), compared with the control cultures (117 fold), treated with cytokines alone. The viability of cells was similar in all culture conditions: 98%, 96%, and 97% for the control cultures, 3′4′-DMF + cytokines and VPA + cytokines, respectively.

Similar to the higher percentage of CD34^+^ cells in 3′4′-DMF and VPA cultures, the fold difference in the CD34^+^ cells was also significantly higher in the 3′4′-DMF + cytokines (27.8 ± 3.4 fold; *P* = 0.03) and VPA + cytokines (34.1 ± 4.1 fold; *P* = 0.004) cultures compared with the cultures with cytokines alone (14.4 ± 2.9 fold). This indicates that both 3′4′-DMF and VPA induced a significantly higher amplification of the CD34^+^ population under normoxia (Figure 2).

**Figure 3:**
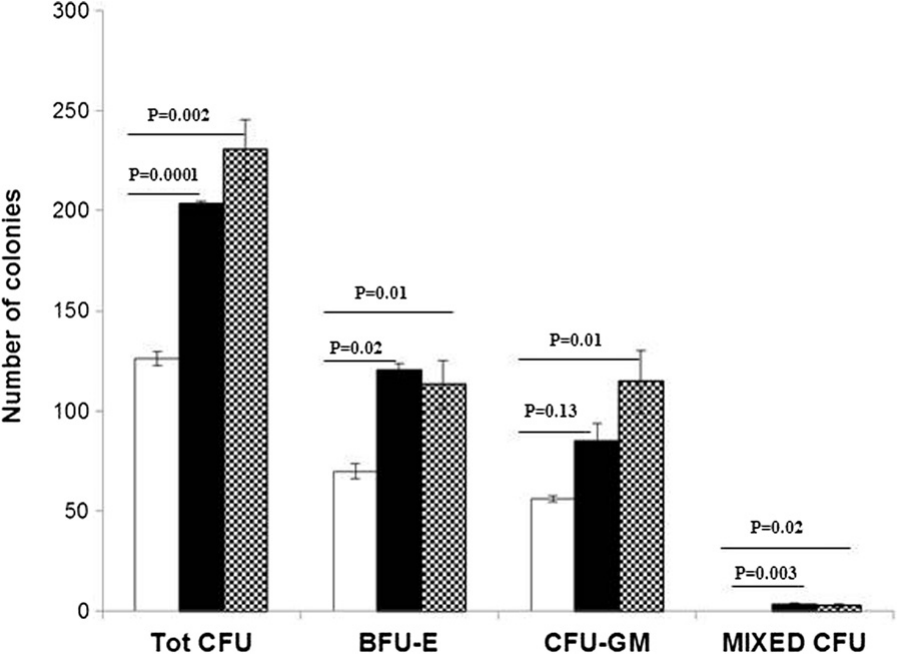
**More committed progenitors (BFU-E, CFU-GM, and CFU-GEMM) in the cultures treated with 3′4′-dimethoxyflavone (DMF) or valproic acid (VPA) compared with the control cultures.** 3′4′-DMF (), VPA (), control (). The columns represent the mean ± SD values from three independent experiments.

Besides the higher proliferation of CD34^+^ cells, cultures with both 3′4′-DMF and VPA resulted in significantly more (*P* < 0.05) primary colonies (plating efficiency) compared with control cultures (Figure 3). In addition, the replating efficiency of the cells treated with 3′4′-DMF (49.5 ± 6.4) or VPA (54 ± 8.5) was higher than that of the control (14.5 ± 6.4) cultures. These assays confirmed that treatment with 3′4′-DMF and VPA helped in preserving the plating efficiency of HSC progenitors.

Based on the promising results, we went ahead to determine the combined effect of 3′4′-DMF and VPA on HSCs. However, no significant difference was observed, in the proliferation or plating efficiency, between the cultures treated with either 3′4′-DMF or VPA and cultures treated with both 3′4′-DMF and VPA, indicating no synergistic effect of the two reagents on HSCs (data not shown).

### Effect of 3′4′-DMF and VPA on the *ex vivo* expansion of CD34^+^ cells under hypoxia (1% O_2_)

**Figure 4:**
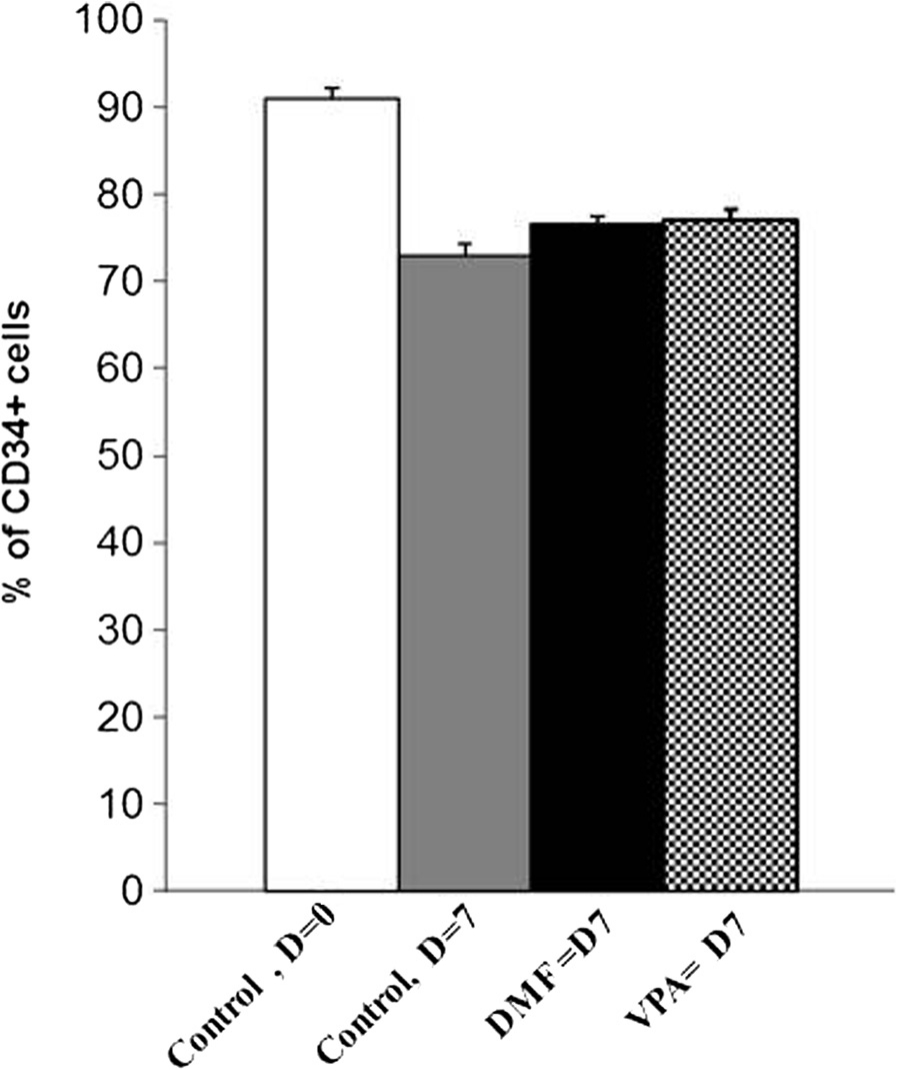
**Effect of 3′4′-dimethoxyflavone (DMF) and valproic acid (VPA) on the frequency of CD34**^**+ **^**cells in the cultures under hypoxia (1% O**_**2**_**).** The percentage of CD34^+^ cells was evaluated before (day 0) and after (day 7) the amplification of cytokine-induced cultures of CD34^+^ cells, maintained at low O_2_ (1%) tension. 3′4′-DMF (),VPA (), control D = 0 (), control D7 (). The columns represent the mean ± SD values from three independent experiments.

In a similar way, the CD34^+^ cells were cultured under hypoxia (1% O_2_) to determine the effect of 3′4′-DMF and VPA on their proliferation and maintaining of CD34 expression. In contrast to the normoxic cultures, a higher expression of CD34 was found in cultures under hypoxia (73% ± 2.4) after 7 days, suggesting that hypoxia plays a role in maintaining the primitive nature of the cells (Figure 4). However, neither 3′4′-DMF (76% ± 2.7) nor VPA (77% ± 1.9) increased the expansion of CD34^+^ cells compared with control (73% ± 2.4) cultures under hypoxia (Figure 4). The percentages of CD34^+^CD90^+^ cells (3′4′-DMF + cytokines = 0.7% ± 0.2, VPA + cytokines = 0.6% ± 0.12, control at day 7 = 0.6% ± 0.17%, and control at day 0 = 0.7% ± 0.2) and CD34^+^CD38^-^ cells (3′4′-DMF + cytokines = 1.5% ± 0.2; VPA + cytokines = 1.6% ± 0.2; control at day 7 = 1.4% ± 0.3, and control at day 0 = 1.6% ± 0.2) did not show any difference in the cultures treated with 3′4′-DMF or VPA in comparison with the control cultures.

**Figure 5:**
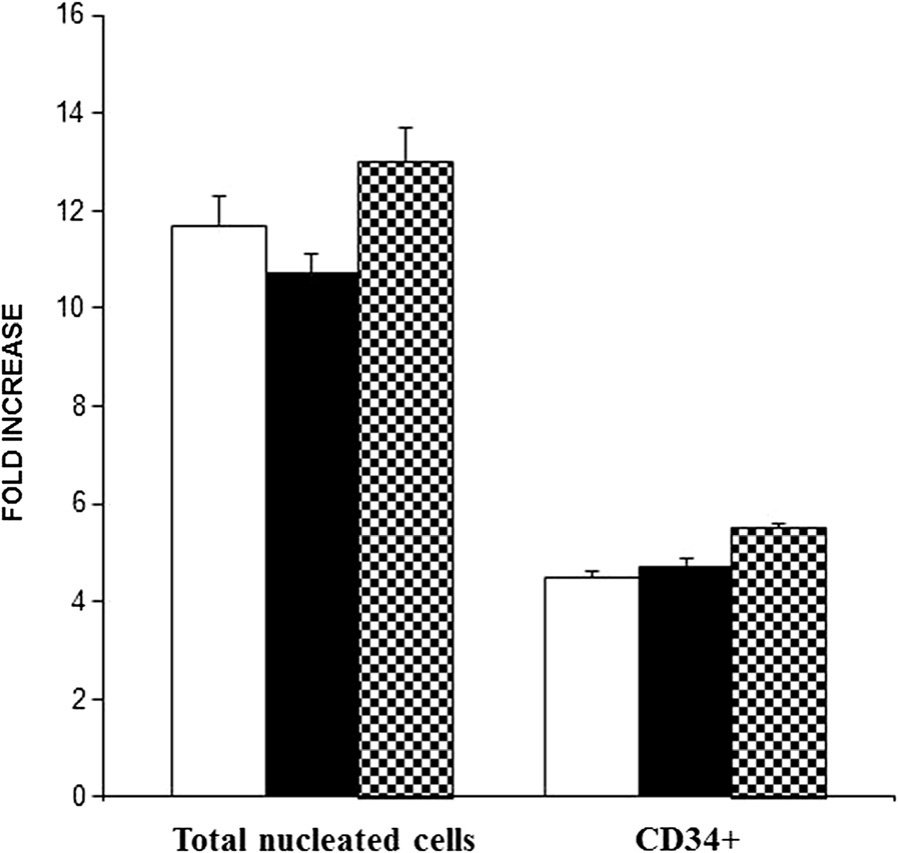
**The effect of 3′4′-dimethoxyflavone (DMF) and valproic acid (VPA) on the amplification of TNC and CD34**^**+ **^**cells, after 7 days of culture under hypoxia, was evaluated as fold increase over the input (day = 0) values.** 3′4′-DMF (), VPA (), control D, 0 (). The columns represent the mean ± SD values from three independent experiments.

Next we investigated the effect of 3′4′-DMF and VPA on the fold increase of TNC and CD34^+^ cells under hypoxia. After 7 days of culture, no difference was observed in the total number of nucleated cells in the control cultures (11.7 ± 0.45 fold) with cytokines alone compared with 3′4′-DMF + cytokines (10.7 ± 0.25 fold) and VPA + cytokines (13 ± 1.1 fold) cultures (Figure 5). The TNC count suggested cell-cycle arrest, which was confirmed by cell-cycle analysis. The cell-cycle analysis revealed an increased number of cells in G_0_ + G_1_ phase (cytokines alone = 67% ± 1.2; 3′4′-DMF + cytokines = 68% ± 2.0; and VPA + cytokines = 67% ± 1.5) and decreased number of cells in S phase (cytokines alone = 26% ± 1.1, 3′4′-DMF + cytokines = 27% ± 1.0; and VPA + cytokines = 27% ± 0.8) for all three culture conditions under hypoxia, when compared with the cells cultured under normoxia (G_0_ + G_1_ phase: cytokines alone = 59.5% ± 1.2; 3′4′-DMF + cytokines = 56.4% ± 1.3; and VPA + cytokines = 57% ± 1.6. S phase: cytokines alone = 32.5% ± 0.9%, 3′4′-DMF + cytokines = 35% ± 1.1; and VPA + cytokines = 35% ± 1.3). In addition, 3′4′-DMF (4.7 ± 0.29 fold) and VPA (5.5 ± 0.37 fold) had no significant effect on the fold increase of CD34^+^ cells in comparison to the control (4.5 ± 0.34 fold) cultures (Figure 5).

**Figure 6:**
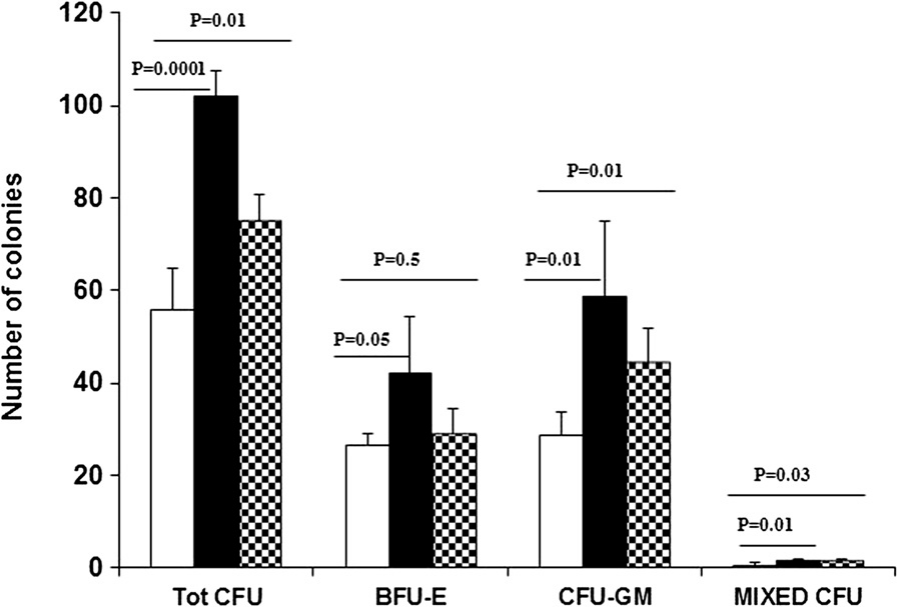
**Effect of 3′4′-dimethoxyflavone (DMF) and valproic acid (VPA) on the committed progenitors of HSCs after 7 days of culture under hypoxia.** A significantly higher number of total colonies and colonies of committed progenitors (CFU-GM and CFU-GEMM) in the cultures treated with 3′4′-DMF or VPA compared with the control cultures. Columns, mean; bars, ±SD. 3′4′-DMF (), VPA (), control (). The columns represent the mean ± SD values from three independent experiments.

Further, we determined the effect of 3′4′-DMF and VPA on the colony-formation assay of HSCs cultured under hypoxia. The HSCs were cultured under hypoxia for 7 days, followed by the CFU assay performed under normoxia. After 14 days, the primary colonies (BFU, CFU-GM, and mixed colonies) were counted. Except for BFU-E, the test revealed significantly more (*P* < 0.05) primary colonies (total CFU, CFU-GM, and mixed CFU) in cultures treated with both 3′4′-DMF and VPA compared with the control cultures (Figure 6).

Similar to normoxia, no synergistic effect of 3′4′-DMF and VPA was observed on HSCs under hypoxia (data not shown).

## Discussion

In this study, we investigated the effect of 3′4′-DMF and VPA on *ex vivo* expansion of HSCs under normoxia and hypoxia. Although the beneficial effects of VPA have been suggested before [11,12], our report is the first, to the best of our knowledge, to show an effect of 3′4′-DMF on HSCs.

Recently, Boitano and co-workers [7] reported the beneficial effects of a purine derivative, SR1, on the expansion of human CD34^+^ cells, from mobilized peripheral blood of donors, by antagonizing the AHR. Although 3′4-DMF has also an effect similar to that of SR1, it has not been previously used in *ex vivo* culturing of HSCs. Treatment of HSC cultures with 3′4′-DMF similarly enhanced the *ex vivo* generation of CD34^+^ cells (27.6 fold compared with 14.5 fold in control) under normoxia (20% O_2_). Thus, our finding is in agreement with Boitano *et al.*[7], who demonstrated expansion of HSCs with another AhR antagonist, SR1.

Contrary to the results obtained under normoxia, no effect on the expansion of CD34^+^ cells was observed in the cultures stimulated with 3′4′-DMF under hypoxia (1% O_2_). However, plating efficiency was higher in the 3′4′-DMF cultures compared with control, under normoxia and hypoxia. This indicates that 3′4′-DMF helps to preserve the hematopoietic progenitors under both normoxia and hypoxia.

The mechanistic studies have shown that 3′4′-DMF acts by directly binding to the AHR, and in turn inhibits the AHR-mediated induction of cytochrome P450 1A1 (CYP1A1) and cytochrome P450 1B1 (CYP1B1). However, the mechanisms of this inhibitory effect of 3′4′-DMF on the expression of these enzymes (CYP1A1 and CYP1B1) remain to be elucidated. The expression of the AHR in HSCs suggests that similar mechanisms (inhibition of AHR-mediated induction of CYP1B1 and CYP1A1) could play a role in the expansion and maintenance of HSCs [14]. However, further studies are required to define the role of CYP1A1 and CYP1B1 as well as the molecular mechanisms involved in these processes. Nevertheless, our data suggest the potential use of a 3′4′-DMF in HSC expansion and maintenance, which requires validation by further studies. Besides, *in vivo* studies, using NOD SCID Gamma (NSG) mouse model, would provide strong supporting evidence to our findings, which, unfortunately, could not be accomplished in the present study because of lack of resources.

The present study has also demonstrated the positive effect of VPA on *ex vivo* expansion of HSCs. Although the potential use of VPA, in the expansion of HSCs, has been documented before [15-18], findings of the present study were different from earlier reports, in terms of percentage of CD34^+^ cells, cell cycle, and CFU. Moreover, nothing is known about the use of VPA on the proliferation, differentiation, survival, and maintenance of the primitive nature of human HSCs under hypoxia. In addition, reported studies used a higher concentration of VPA (1 m*M*) in the cultures [15-18]. The concentration of VPA (0.065 m*M*) used in our study was very much lower in contrast to the concentrations used by Felice *et al.* (1 m*M*) [14], Bug *et al.* (0.9 m*M*) [16], and Burba *et al.* (2.5 m*M*) [18], and in addition, we also tested the effect of VPA under hypoxic conditions. All these studies reported an expansion of CD34^+^ in human HSC cultures after 7 days. On the contrary, in the present study, the percentage of CD34^+^ cells decreased after 7 days of culture compared with input cells at day 0. However, the percentage of CD34^+^ cells (20.3%) in our study was comparable to the results obtained by Seet *et al.*[17], which could again be attributed to the higher concentrations of VPA used in other studies [15,16,18].

The present study showed that exposure to VPA enhances the number of CFUs in HSCs, in contrast to the results obtained by De Felice *et al.*[15], in which the number of CFUs decreased in the cultures stimulated with VPA. Moreover, contrary to De Felice *et al.*, in which authors showed that VPA lengthens the duration of cell cycle, with most of the cells in G_0_/G_1_ phase [15], we did not observe any cell-cycle arrest.

Notably, our data showed a 19.5-fold increase in CD34^+^ cells after 7 days in VPA cultures compared with control cultures (34 fold versus 14.5 fold), which was in agreement with the study by De Felice *et al*. [15]. Moreover, both the studies demonstrated an increase in the replating efficiency of the cells in VPA + cytokines cultures compared with the control cultures.

Surprisingly, our data is in agreement with the results obtained by Bug *et al*., [16] in terms of CFU and cell-cycle analysis. In this study, authors exposed the murine Sca^+^/lin^-^ HSCs to VPA and found that VPA increased the number of CFUs and the replating efficiency. Also in contrast to the control, VPA-treated cultures had increased percentages of cells in the S phase. These results suggest that exposure of human HSCs to low concentration of VPA could have an effect similar to that of the treatment of murine HSCs with high VPA concentrations, which points toward the potential possibility of different biochemical pathways or different signaling molecules involved in the two species.

In contrast to the normoxic cultures, VPA did not stimulate the enhancement of proliferation of TNC or CD34^+^ cells under hypoxia. However, VPA significantly increased the plating efficiency of HSCs compared with control untreated cultures. Hence, as in 3′4′-DMF, VPA could play a role in preserving the progenitors, under both normoxia and hypoxia.

## Conclusions

In conclusion, our study suggests a positive effect of 3′4′-DMF on human hematopoietic stem cells. However, it warrants further studies to validate our findings as well as to explore further the potential benefits of 3′4′-DMF, either alone or in synergy with other agents, in HSC biology.

Further studies are required to clarify the use of VPA, especially with same cell source, similar culture conditions, and cytokine cocktail, to find an optimal VPA concentration for *ex vivo* expansion and maintenance of human HSCs. Moreover, the differences in mouse and human HSCs, when exposed to VPA, should be investigated further before using the former as *in vivo* model for the study.

## Abbreviations

3′4′-DMF: 3′4′-dimethoxyflavone; CFU: Colony-forming unit; HSC: Hematopoietic stem cell; VPA: Valproic acid.

## Competing interests

None of the authors of this manuscript has any relevant conflicts/interests to declare.

## Authors’ contributions

KK helped in designing the study, designed the experiments, carried out the cell-culture experiments and colonogenic experiments, performed statistical analysis, and drafted the manuscript. MRM helped in designing the experiments, isolated CD34^+^ cells, carried out the FACS and cell-cycle experiments, and contributed in the discussion and interpretation of results. GK provided CD34^+^ cells and contributed in the discussion and interpretation of results. JKK conceived the study and helped in the design and coordination of the study and interpretation of results. All the authors read and approved the final manuscript.

## Authors’ information

KK is a PhD in molecular genetics and worked for 1 year on this project as a postdoctoral researcher at Department of Immunology and Transfusion Medicine, Oslo University Hospital, Oslo, Norway. MRM is a PhD and works as a researcher at Department of Cellular Therapy, Oslo University Hospital, Ullevål, Oslo, Norway.

GK is a PhD, working as Head of the Department of Cellular Therapy, Oslo University Hospital, Radiumhospitalet, Oslo, Norway.

JKK has had a position as Senior Consultant Physician (since 1996) and Professor of Transfusion Medicine (since 2004). His presents address is Department of Clinical Immunology and Transfusion Medicine, University and Regional Laboratories Region Skåne, Lund, Sweden.
